# Pathogenic germline variants in patients with breast cancer: conversations across generations, practices and patients’ attitude

**DOI:** 10.3389/fgene.2023.1194075

**Published:** 2023-10-18

**Authors:** Hikmat Abdel-Razeq, Rawan Mustafa, Sarah Abdel-Razeq, Hala Abu-Fares, Sama Al Masri, Rana Damsees, Mariam El-Atrash, Shatha Elemian, Mais Alkyam, Khawlah Ammar, Rayan Bater, Marah Kderat, Abdulrahman Alhajahjeh

**Affiliations:** ^1^ King Hussein Cancer Center, Department of Internal Medicine, Amman, Jordan; ^2^ School of Medicine, The University of Jordan, Amman, Jordan; ^3^ King Hussein Cancer Center, The Office of Scientific Affairs and Research, Amman, Jordan

**Keywords:** breast cancer, family communication, germline genetic testing, *BRCA*, cascade testing

## Abstract

**Background:** Breast cancer susceptibility genes such as *BRCA1*, *BRCA2*, *PALB2*, *CHEK2* and many others are increasingly recognized among our patient population. In addition to their impact on treatment decisions of tested patients themselves, identifying at-risk family members offer opportunities for cancer preventive measures.

**Methods:** This is an observational cross-sectional study of adult breast cancer patients with positive breast-cancer-susceptibility germline variants who received treatment at our institution. Patients with variants of uncertain significance (VUS), or who refused to give consent, were excluded. The data was collected from an eligible sample of breast cancer patients using a structured questionnaire developed by the study team and tested for validity and reliability, as well as a clinical chart review form. Patients were invited to participate in the study during their scheduled oncology clinics visit.

**Results:** 169 patients were enrolled, including 42 (24.9%) with pathogenic/likely pathogenic (P/LP) *BRCA1* variants, 84 (49.7%) with *BRCA2* and 43 (25.4%) with non-BRCA variants. All patients were female and the mean age was 45 ± 9.9 years. Among 140 eligible patients, 104 (74.3%) underwent prophylactic mastectomy, while 79 (59.0%) of 134 eligible patients had prophylactic bilateral salpingo-oophorectomy (BSO). Results were communicated with family members by majority (*n* = 160, 94.7%), including 642 first degree female relatives, and 286 (44.5%) of them have taken no action. Fear of positive test results, cost of testing, unwillingness to undergo preventive measures, and social stigma were cited as barriers to genetic testing in 54%, 50%, 34% and 15%, respectively.

**Conclusion:** Risk-reducing interventions including mastectomy and BSO were carried by majority of patients with P/LP variants. However, though the rate of communication of genetic testing results with family members was high, proper preventive measures were relatively low. Cost and fear of cancer diagnosis, were the leading causes that prevented cascade testing in our cohort.

## 1 Introduction

Breast cancer is the most commonly diagnosed cancer among women worldwide (Arnold et al., 2022). P/LP variants in cancer-predisposing genes like *BRCA1* and *BRCA2* account for 5%–10% of breast cancer cases (Foulkes, 2008; Larsen et al., 2014). Since the introduction of both *BRCA1* and *BRCA2* almost 30 years ago, several variants in other genes were identified including *CHEK2, PALB2, ATM, TP53* and many others. In a meta-analysis of 10 eligible studies, the estimated mean cumulative risk for developing breast and ovarian cancers by age 70 for carriers of the *BRCA1* variant is 57% and 40%, respectively, whereas the risk for carriers of the *BRCA2* variant is a little lower at 49% and 18%, respectively (Chen and Parmigiani, 2007). The extent to which other P/LP variants are associated with breast cancer susceptibility varies significantly (Seal et al., 2006; Rahman et al., 2007). Prophylactic mastectomy is sometimes considered for other genes, and each case must be evaluated on its own merits based on specific gene involved, international guidelines, patient’s family history, clinician’s professional judgment and most importantly patient’s wishes (NCCN Clinical Practice Guidelines in Oncology, 2023).

To better serve our patients, policymakers need information regarding the pattern and prevalence of ancestry-specific cancer-predisposing P/LP germline variants (PGV). Information about hereditary breast cancer rates in Arab countries is limited and very variable. We previously reported the distribution and frequency of germline *BRCA1* and *BRCA2* variants in high-risk Jordanian breast cancer patients, chosen in accordance with international guidelines. The median age of the 517 participants was 39 years (range: 19–78 years). In total, 72 (13.9%) patients had P/LP *BRCA1* or *BRCA2* variants and 53 (10.3%) had variants of uncertain significance (VUS) (Abdel-Razeq et al., 2020). More recently, we reported our experience in conducting a multi-gene panel testing on 1,310 patients tested as per the National Comprehensive Cancer Network (NCCN) guidelines. Among the whole group, 184 (14.0%) patients had P/LP variants; only 90 (48.9%) were in *BRCA1* or *BRCA2*, while 94 (51.9%) others had P/LP variants in other genes; mostly in *APC*, *TP53*, *CHEK2* and *PALB2* (Abdel-Razeq et al., 2023).

Several studies were conducted to evaluate patients’ attitude and acceptance rates of genetic testing and to understand the psychological and behavioral impact of breast cancer susceptibility genetic test results. Members of a positive family should be aware that they have a higher risk of developing breast, ovarian, and other cancers (Lieb et al., 2018). Relatives who may be carriers may benefit from monitoring and preventative measures (Domchek et al., 2006; Domchek et al., 2010). Maintaining open lines of communication within the family is crucial for informing those at-risk of being carriers of the disease. Though rate of communicating P/LP germline variants with family members can be high, the rate of cascade genetic testing is much lower. People’s reactions to the results of genetic testing, the prospect of passing on an altered gene to their offspring, and the resulting anxiety and uncertainty can be very stressful for the patients and their family members (Landsbergen et al., 2005; Finlay et al., 2008a). In one study, 115 people with a P/LP *BRCA1* or *BRCA2* variants were asked to fill out a survey about whether they had informed their at-risk relatives about their variant and whether they had received genetic testing as a result. Counseling and testing for at-risk relatives were provided at no cost and with complete privacy. The study found a very high rate of disclosure (95%) among first-degree relatives, but the uptake of genetic testing among these individuals reached 60%, suggesting that factors other than cost are involved in the decision to perform cascade testing (Reid and Pal, 2020).

The results of genetic testing and the adoption of preventative measures are received and understood differently by people from different cultural backgrounds. Although the prevalence of PGV is similar among non-Caucasians, the prevalence of VUS is higher, which may impact the ability of non-Caucasian patients to interpret their genetic results (Abdel-Razeq et al., 2022). Little is known about the experiences of non-Western women who have undergone germline breast cancer susceptibility testing, including whether they discuss the testing or the results with their spouse, family, and/or close relatives and the potential effects this may have on their relationships. The findings of this study can be used by genetic counseling services and the general public to emphasize the importance of communicating test results to high-risk close relatives.

## 2 Materials and methods

### 2.1 Study design

This was an observational, cross-sectional study of adult Jordanian breast cancer patients with P/LP variants conducted between 2018 and 2021.

### 2.2 Study participants

Eligible participants were adults above 18 years of age, diagnosed with breast cancer with positive PGV confirmed by molecular susceptibility testing, and counseled at our institution. Patients with VUS or those who refused to provide consent were excluded.

### 2.3 Recruitment and procedure

We utilized our database of all breast cancer patients tested as per the NCCN guidelines for PGV. To identify potential participants, those meeting eligibility criteria were invited to participate during their visit to their oncologist clinic, or during Tele-Clinic using different meeting platforms. A brief introduction was given about the study’s purpose and requirements, and participants were clearly informed that completing the questionnaire would depend on their consent to participate. This study was approved by the institutional review board (IRB) of the King Hussein Cancer Center.

Genetic testing was performed using a peripheral blood sample. Whole gene sequencing, deletion, and duplication analysis of all coding exons, ±20 base pairs of flanking introns was performed and interpretated at Invitae Corp. (San Francisco, United States) as previously described (Nykamp et al., 2017). Variants were classified as negative, pathogenic/likely pathogenic (positive) and variant of uncertain significance (VUS).

### 2.4 Questionnaire

We developed a two-part, 20-item survey that included socio-demographic factors, such as age, number of children, date of diagnosis, marital status, employment, and educational attainment. Part II assessed personal cancer history, history of cancer in first- and second-degree relatives, family history of *BRCA1/2* testing, genetic test results, family notification of testing, and subsequent testing by relatives. We measured the perceived importance of testing, while communicating the results with relatives led to preventive actions from their side. These items were developed via literature review.

### 2.5 Data analysis

Descriptive statistics were used to analysis the study data, continuous variables were presented as mean and standard deviation while categorical variables were presented as frequencies and percentages. Chi square test was used to measure the association between *BRCA1/2* and non-BRCA carriers in terms of preventive surgeries. *p*-value at ≤0.05 was considered significant. The data was analyzed using the Statistical Package for Social Science (SPSS version 28).

## 3 Results

### 3.1 Patients’ characteristics

Among our pool of patients with P/LP germline variants, 169 patients with breast cancer agreed to be enrolled and returned the study questionnaire. The mean age ± STD of the participants was 45 ± 9.9 (range, 27–77) years and all were female. Most (*n* = 127, 75.1%) of the patients were married, have children (*n* = 125, 74.0%), received college education (*n* = 96, 56.8%) and 49.7% have at least one first-degree relative with breast cancer, Table 1.

**TABLE 1:** Patients characteristics (*n* = 169).

Characteristics		Number	Percentage
Marital status	Married	127	75.1
	Single	20	11.8
	Widowed	11	6.5
	Divorced/Separated	8	4.7
Level of education	High school and below	68	40.2
	College/University	96	56.8
Had children	Yes	125	74.0
	No	44	26.0
Breast cancer susceptibility genes	*BRCA1*	42	24.9
	*BRCA2*	84	49.7
	Non-BRCA genes	43	25.4
Family history of breast cancer in 1st degree relative	Yes	84	49.7
	No	85	50.3

### 3.2 Risk-reducing surgeries

**FIGURE 1:**
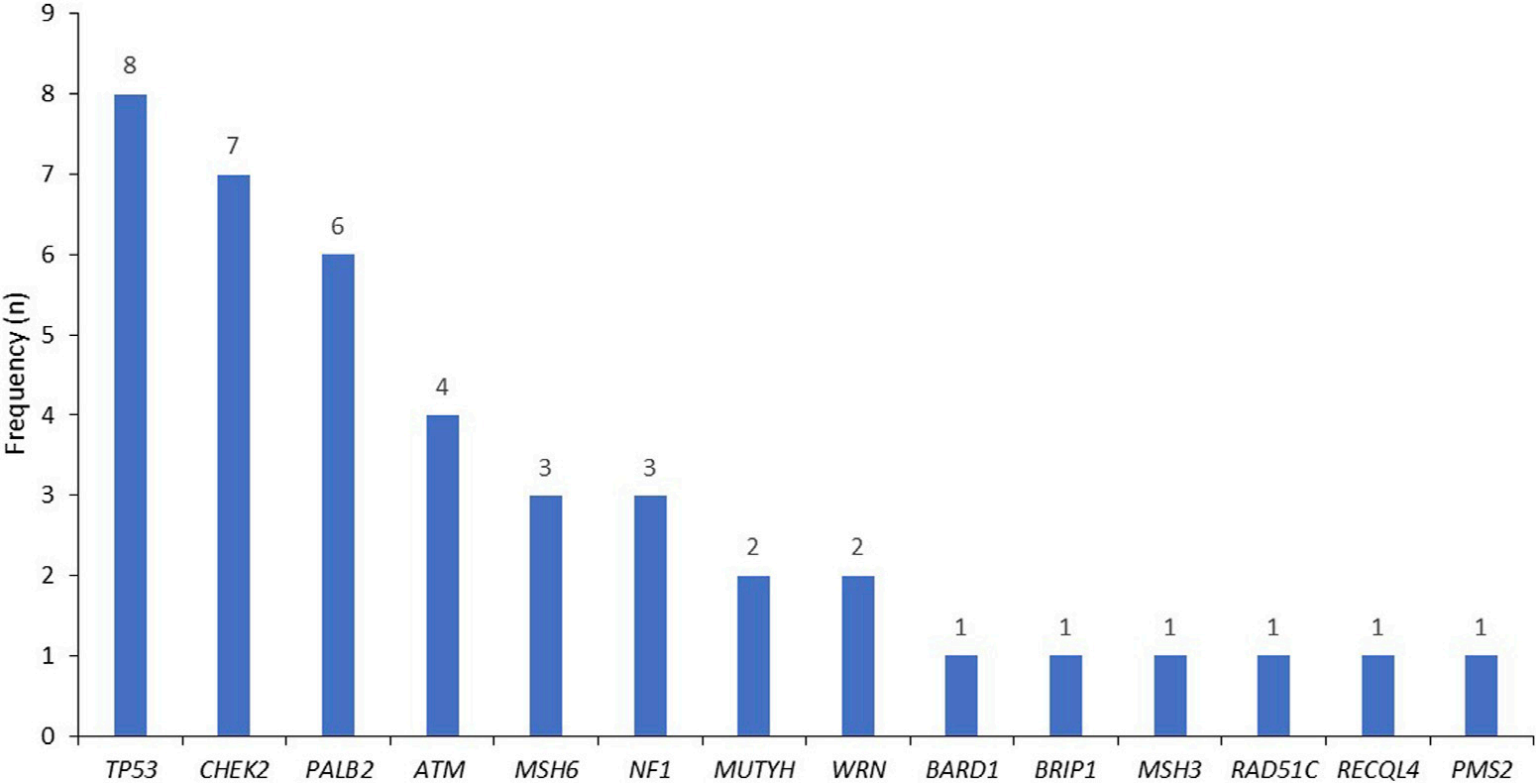
Frequency of pathogenic/likely pathogenic variants (non-*BRCA1/2*).

Among the study patients, 42 (24.9%) had P/LP *BRCA1,* 84 (49.7%) in *BRCA2,* and 43 (25.4%) others had non-BRCA genes, mostly in *TP53, CHEK2, PALB2* and *ATM,* Figure 1.

Among 140 eligible patients, 104 (74.3%) had undergone prophylactic bilateral mastectomies as recommended by their treating physicians, while 79 (59.0%) of 134 eligible patients had undergone prophylactic bilateral salpingo-oophorectomy (BSO). Such risk-reducing surgeries were performed significantly more among those with *BRCA1* and *BRCA2* carriers compared to those with non-BRCA variants, Table 2.

**TABLE 2:** Uptake of preventive surgeries by *BRCA1/BRCA2* carriers versus non-BRCA carriers.

Risk-reducing surgery	*BRCA1/BRCA2* (*n* = 126)	Non-BRCA (*n* = 43)	*p*-value
Prophylactic contralateral breast surgery advised	125 (99.2%)	15 (34.9%)	<0.001
Prophylactic bilateral mastectomies performed	92 (73.0%)	12 (27.9%)	<0.001
Prophylactic BSO advised	126 (100%)	8 (18.6%)	0.002
Prophylactic BSO performed	68 (54.0%)	11 (25.6%)	<0.001

BSO, Bilateral salpingo-oopherctomy.

### 3.3 Communicating results with family members

The questionnaire included questions on the disclosure of results to family members; 160 (94.7%) patients informed their family members of the results, and 133 (83.1%) had done so immediately after getting the results. However, only 103 (60.9%) stated that they have done so following a physician’s advice. Interestingly, those with *BRCA1/BRCA2* were more likely to inform their family members compared to those with non-BRCA P/LP variants; 94.4% (*n* = 119) compared to 81.4% (*n* = 35) among those with non-*BRCA1/BRCA2* germline variants, *p* = 0.024.

Majority (*n* = 124, 73.4%) of patients believe that they themselves should inform their family members, while 27 (16.0%) believe it is the physician’s duty to do so. Results of genetic testing were communicated with spouses by 118 (69.8%) patients, while 11 (6.5%) opted not to involve their spouses (Table 3).

**TABLE 3:** Genetic testing communication (*n* = 169).

Communication elements		Number (%)
Have you informed your close family members of the results?	Yes	160 (94.7%)
	No	9 (5.3%)
Have you been advised by your physician about the necessity of informing at-risk family members?	Yes	103 (60.9%)
	No	66 (39.1%)
Who, do you think, is responsible of informing the family?	The patient	124 (73.4%)
	The physician	27 (16.0%)
	The genetic counsellor	6 (3.6%)
	Others	12 (7.1%)
Was your social status affected by genetic testing results?	Yes	32 (18.9%)
	No	137 (81.1%)
Have you informed your spouse of genetic testing results?	Yes	118 (69.8%)
	No	11 (6.5%)
	No answer	40 (23.7%)

Patients cited many reasons that encouraged them to inform their relatives. In addition to the desire of preventing cancer, many (*n* = 36, 22.5%) stated that they did so because they needed family support and help in making their own medical decisions. For the minority who did not inform their families (*n* = 9, 5.3%), many (*n* = 7) were worried of the social consequences of the results, including possible negative effect on the marriage of their offspring.

### 3.4 Patients’ reaction to positive tests

Patients reported different emotional responses to the results of the genetic testing: 73 (43.2%) experienced anxiety, 45 (26.6%) were fearful of the future and the decisions of childbearing, 96 (56.8%) were worried about their children carrying the disease*,* while 36 (21.3%) reported they felt comforted because they can take preventive measures to protect themselves and their family members, and only 9 (5.3%) were worried about health insurance coverage and difficulties obtaining health insurance.

### 3.5 Relatives’ reactions

**FIGURE 2:**
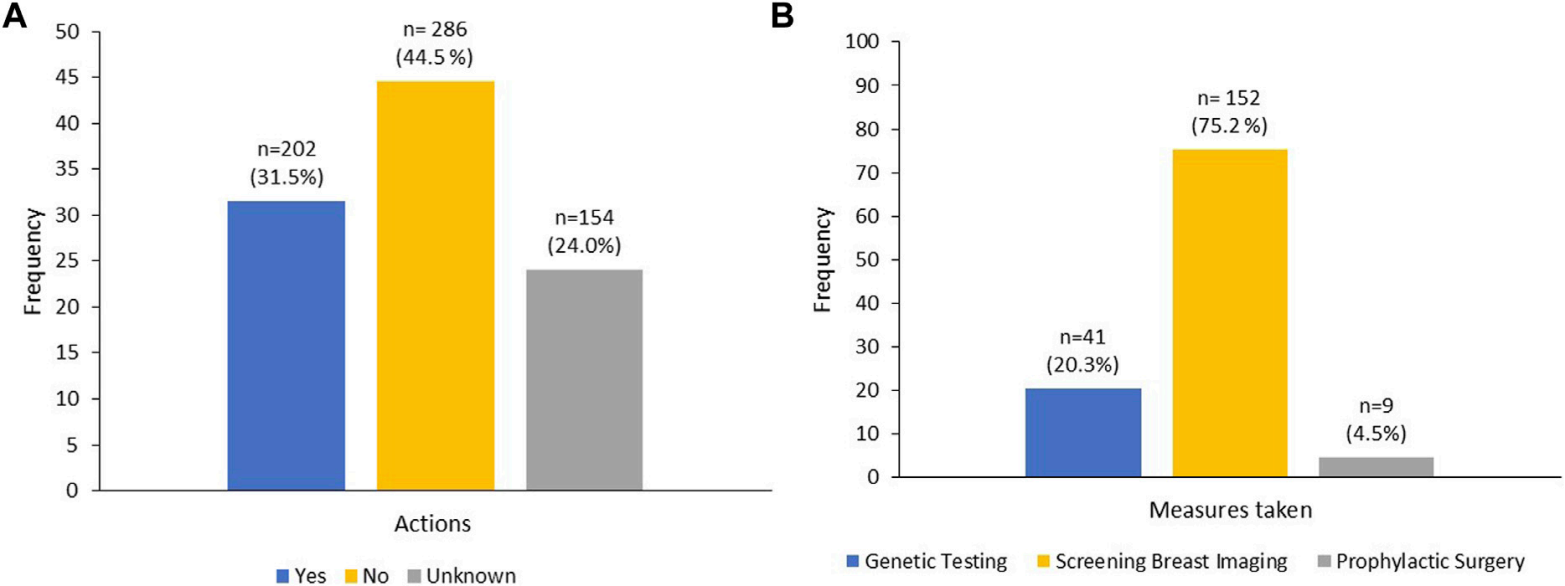
Percentage of informed 1st degree female family members who had taken action **(A)** and measures taken **(B)**.

**FIGURE 3:**
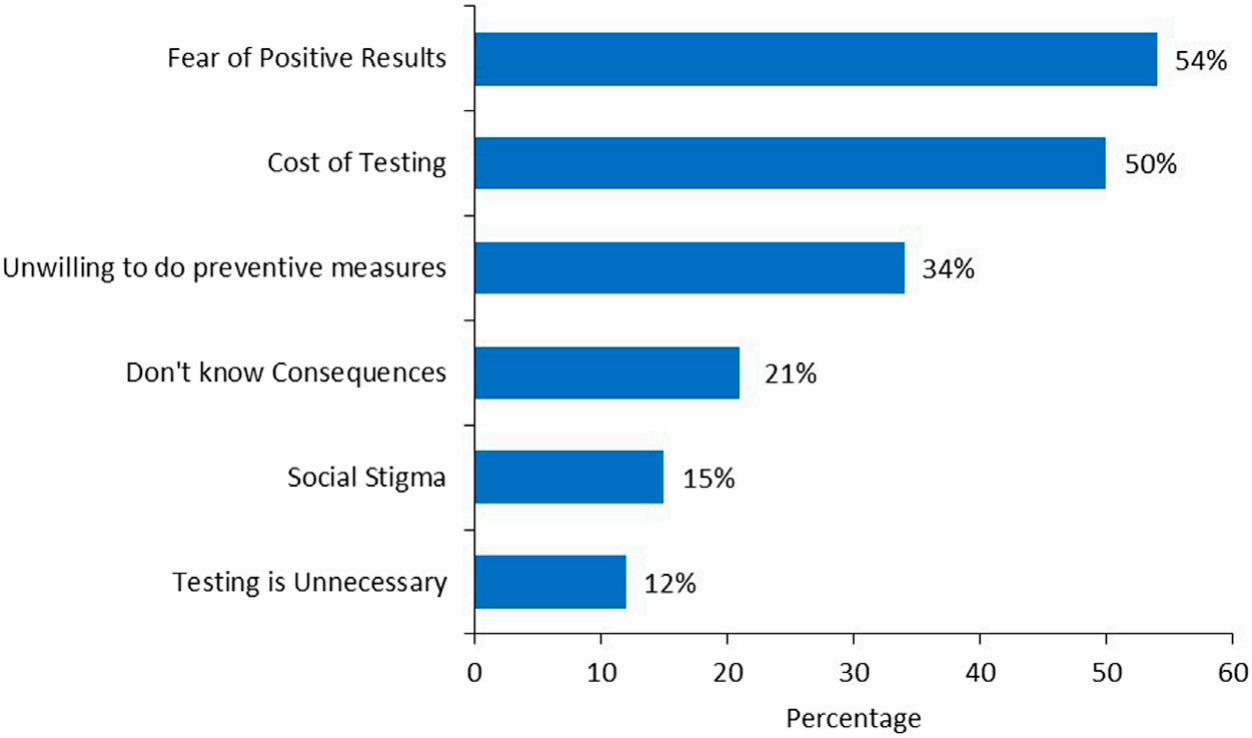
Barriers of cascade testing (percentage).

A total of 1,396 relatives were informed by our 169-patient cohort, i.e., an average of 8–9 family members per patient; of these, 642 (46.0%) were first degree female relatives (mother, sister, and daughter), 286 (44.5%) of them had not taken any action based on the test results. Among the 202 (31.5%) who took actions, 41 (20.3%) underwent genetic testing and only 9 (4.5%) patients underwent prophylactic mastectomy. However, 152 (75.2%) underwent screening breast imaging, either by ultrasound (US) or mammography, Figures 2A, B. Several reasons were cited by family members that prevented them from acting on test results including, fears of positive results (54%), cost of testing (50%) and unwillingness to undergo preventive measures (34%), while fears of social stigma of carrying a cancer gene was a factor in 26 (15%), Figure 3.

## 4 Discussion

The open communication of serious health-related issues present within families is a complex process. This process is even more intricate when it is linked to inherited disorders such as familial cancer, where being a mutation carrier could implicate a life threatening situation. Such conversations are furthermore hindered in communities where a cancer diagnosis is kept in secret and not openly discussed with family members, especially in non-Western societies.

Since the establishment of cancer genetics, initially as a research program, then as a clinical service, we have witnessed major improvement in patients’ willingness for germline genetic testing and communicating results to close family members. The benefits of genetic testing are currently being discussed with each patient through consultation with genetic healthcare providers, as it could potentially impact their treatment regime and/or risk-reducing interventions. Simultaneously, the importance of communicating positive test results to at-risk family members through the index patient are accentuated, as the healthcare providers do not make contact with relatives.

Family communication was high in our study cohort, with 95% of variant positive patients disclosing their status to family members. Of these, 83% had done so immediately after receiving their results. This gesture indicated that patients are fully aware of the social and medical consequences and impact the genetic test result. This is in-line with Western literature (MacDonald et al., 2007; Finlay et al., 2008b; Lemke et al., 2022).

Data from our cohort revealed that those with *BRCA1/BRCA2* P/LP variants were more likely to inform their family members than those with non-BRCA variants. Previous studies had shown similar results (Hughes et al., 2002; Costalas et al., 2003). This could be due to patients’ and healthcare professionals’ unfamiliarity with pathogenic variants in other high-impact non-BRCA genes. In the meantime, until more extensive epidemiological and clinical studies demonstrate the benefit of screening and testing for lower frequency P/LP variants, experts recommend focusing population-based efforts on high penetrance genes, such as *BRCA1* and *BRCA2*, while keeping the option of individualized precision preventive measures based on common genetic and non-genetic risk factors (Turnbull et al., 2018).

Our study also emphasizes patient-physician communication; only 61% of physicians advised patients to communicate the genetic test results with their relatives at potential risk. It is widely assumed that it is the patient’s moral duty to inform their at-risk relatives, and that the role of healthcare professionals, including physicians, is to assist patients in fulfilling this duty. However, Grill and Rosén in a recent study argued that if a patient’s genetic data reveals a P/LP variant in a high-penetrant disease-causing gene, and if effective preventive measures are available, then healthcare providers with this information, have a moral obligation to investigate whether the patient has any at-risk relatives, and if so, to ensure that the information is made available to them (Grill and Rosén, 2021).

Although a high number of relatives were informed about the result of the patient’s genetic tests, 44% of first-degree relatives who were informed did not take any action, with only 20.3% opting for testing. Such results contrast data published among Western patients by Cheung et al. (2010), in which 75% of *BRCA*-positive participants reported that at least one relative pursued genetic testing. Such disparity can be attributed to the lack of knowledge of screening and risk reduction recommendations among our patients (Cheung et al., 2010).

The barriers highlighted by our cohort were expected and anticipated. Some of these barriers can be managed and improved including the cost of testing. Many diagnostic laboratories do offer significant discounts or even free testing for at-risk family members, if requested soon after testing the index case. Our results and available data, should convince insurance companies and decision makers, that germline genetic testing can be cost-effective, too. Additionally, proper counselling of patients and relatives, by experienced staff, should lessen their fears of a potential cancer risk.

## 5 Conclusion

Although the rate of communicating genetic test results with at-risk family members was high, there is a lack of awareness regarding proper preventive measures to decrease the risk of breast and other cancers. Cost and fear of a potential cancer risk were the leading causes that prevented cascade testing in our cohort.

## Data Availability

The raw data supporting the conclusion of this article will be made available by the authors, without undue reservation.
